# Mutations in a Novel Cadherin Gene Associated with Bt Resistance in *Helicoverpa zea*

**DOI:** 10.1534/g3.120.401053

**Published:** 2020-03-16

**Authors:** Megan L. Fritz, Schyler O. Nunziata, Rong Guo, Bruce E. Tabashnik, Yves Carrière

**Affiliations:** * Department of Entomology, University of Maryland, College Park, MD 20742; † Department of Entomology, University of Arizona, Tucson, AZ 85721

**Keywords:** Cadherin, Cry1Ac, genome scanning, resistance, selection

## Abstract

Transgenic corn and cotton produce crystalline (Cry) proteins derived from the soil bacterium *Bacillus thuringiensis* (Bt) that are toxic to lepidopteran larvae. *Helicoverpa zea*, a key pest of corn and cotton in the U.S., has evolved widespread resistance to these proteins produced in Bt corn and cotton. While the genomic targets of Cry selection and the mutations that produce resistant phenotypes are known in other lepidopteran species, little is known about how selection by Cry proteins shape the genome of *H. zea*. We scanned the genomes of Cry1Ac-selected and unselected *H. zea* lines, and identified twelve genes on five scaffolds that differed between lines, including *cadherin-86C* (*cad-86C*), a gene from a family that is involved in Cry1A resistance in other lepidopterans. Although this gene was expressed in the *H. zea* larval midgut, the protein it encodes has only 17 to 22% identity with cadherin proteins from other species previously reported to be involved in Bt resistance. An analysis of midgut-expressed cDNAs showed significant between-line differences in the frequencies of putative nonsynonymous substitutions (both SNPs and indels). Our results indicate that *cad-86C* is a likely target of Cry1Ac selection in *H. zea*. It remains unclear, however, whether genomic changes at this locus directly disrupt midgut binding of Cry1Ac and cause Bt resistance, or indirectly enhance fitness of *H. zea* in the presence of Cry1Ac by some other mechanism. Future work should investigate phenotypic effects of these nonsynonymous substitutions and their impact on fitness of *H. zea* larvae that ingest Cry1Ac.

Transgenic crops are extensively used for the management of both insect and plant pests worldwide, which places extraordinary pressure on pest species to adapt (Tabashnik and Carrière 2017; Gould *et al.* 2018). The first generation of commercially available transgenic crops included corn and cotton bioengineered to produce a single crystalline (Cry) protein from the soil-dwelling bacterium, *Bacillus thuringiensis* (Bt) (Gould 1998). The primary targets of these Cry proteins were difficult-to-manage lepidopteran larvae (Roush 1997). When fed upon Cry-producing plant tissue, larvae often experience significant reductions in growth and survivorship (Clark *et al.* 2000; Tabashnik *et al.* 2000; Ali *et al.* 2006; Liu *et al.* 2010; Pardo-López *et al.* 2013). Not all targeted lepidopteran species are highly susceptible to Cry proteins, however. Species with low inherent susceptibility are expected (Gould 1998; Tabashnik *et al.* 2004; Carrière *et al.* 2015) and do evolve resistance to Bt crops faster than species with high susceptibility (Tabashnik and Carrière 2017). Accordingly, there is growing concern over the number of species that have evolved significant resistance to Bt crops producing Cry proteins (Tabashnik and Carrière 2017, 2019; Gould *et al.* 2018; USEPA 2018).

*Helicoverpa zea* is one lepidopteran species targeted by Bt crops with low inherent susceptibility to Cry proteins (Stone and Sims 1993; Ali *et al.* 2006; Sivasupramaniam *et al.* 2008). A major pest of both corn and cotton in the U.S., *H. zea* has evolved resistance to several Cry proteins (Welch *et al.* 2015; Dively *et al.* 2016; Reisig *et al.* 2018; Kaur *et al.* 2019; Yang *et al.* 2019). While some efforts have been made to understand the mechanisms underlying Cry resistance in *H. zea* (Caccia *et al.* 2012; Zhang *et al.* 2019), we still know little about how selection by Cry proteins has shaped genotype frequencies in this important pest.

Work in other Cry-resistant Lepidoptera provides clues as to which genes are potential targets of selection in *H. zea*, however. When a larva ingests Cry proteins, these toxins bind to receptors in the midgut, which lead to pore formation and lethal midgut cell death (Pardo-López *et al.* 2013). Disruption of toxin binding to larval midgut receptors is the most common mechanism of resistance (Peterson *et al.* 2017). Mutations that alter the coding sequence or reduce expression of Cry1A-binding cadherin proteins are associated with resistance to Cry1A toxins in several major lepidopteran pests (Gahan *et al.* 2001; Morin *et al.* 2003; Xu *et al.* 2005; Fabrick *et al.* 2014; Zhang *et al.* 2017; Wang *et al.* 2018).

Here, we used a genome scanning approach to compare previously described Cry1Ac-selected and unselected lines of *H. zea* (Brévault *et al.* 2013, 2015; Orpet *et al.* 2015a, 2015b; Welch *et al.* 2015; Carrière *et al.* 2018a). We identified five regions of the genome showing signatures of selection, one of which includes a novel gene from the cadherin family, which has been shown to comprise genes associated with Cry resistance in other lepidopteran species (Gahan *et al.* 2001; Fabrick *et al.* 2014; Zhang *et al.* 2017). We compared the predicted protein sequence of the novel cadherin with cadherins involved in Bt resistance in other lepidopteran species, and analyzed both the midgut expression patterns and predicted amino acid sequence differences at this gene for selected and unselected *H. zea* lines. Finally, we discuss the possible functional roles of this gene in *H. zea* resistance to Cry proteins.

## Materials and Methods

### Insect material

We conducted our screen for candidate genes associated with Bt resistance in two laboratory-reared lines of *H. zea* that differed in susceptibility to the Bt toxin Cry1Ac. The lines were founded with 180 larvae collected in Georgia from Cry1Ab corn in 2008 (Brévault *et al.* 2013). F_1_ progeny from field-collected individuals gave rise to the GA line, which was reared on wheat-germ diet and was unexposed to Bt toxins. After two generations of laboratory rearing, we used a subset of insects from GA to initiate the GA-R line, which was selected for resistance to Cry1Ac. From 2008 to 2012, each line was reared with *ca*. 600 individuals per generation, with approximately 11 generations per year. Between 2008 and 2010, GA-R was selected with Cry1Ac over 9 non-consecutive generations as described in Brévault *et al.* (2013). This yielded 10-fold resistance to Cry1Ac and significantly higher survival on Cry1Ac and Cry1Ac + Cry2Ab cotton in GA-R relative to GA (Brévault *et al.* 2013). From 2010 to 2012, GA-R was selected again with Cry1Ac over 10 non-consecutive generations. In 2012, GA-R was crossed to GA to generate a new GA-R line, and the new GA-R and original GA lines were split into two subsets, each reared with *ca*. 600 individuals per generation (Orpet *et al.* 2015a). The two subsets of each line were crossed every second or third generation to generate two new subsets (Orpet *et al.* 2015a). The new GA-R was selected for seven non-consecutive generations with Cry1Ac, which yielded 14-fold resistance to Cry1Ac in GA-R relative to GA (Orpet *et al.* 2015a) and significantly higher survival on Cry1Ac + Cry2Ab cotton in GA-R than GA (Carrière* et al*. 2019). Between 2012 and 2016, GA-R was selected for an additional 25 non-consecutive generations with Cry1Ac, using the methods described in Carrière *et al.* (2018a). Male pupae sampled for genomic analysis were from generations F52 for GA-R (n = 5) and F72 for GA (n = 5) reared in October 2016.

### DNA isolation and whole genome sequencing

We extracted whole genomic DNA from 5 individuals per line using a Qiagen DNEasy Blood and Tissue Kit (Qiagen, Inc., Valencia, CA, USA) following the mouse tail protocol with some modifications. For each individual, DNA was extracted from half of a pupa placed into a 2 mL microcentrifuge tube, containing 180 *μ*L ATL buffer and 20 *μ*L proteinase K. A sterile ceramic bead was added to each tube, and the capped microcentrifuge tubes were placed on a Benchmark Benchmix vortex with a multi-head attachment (Genesee Scientific Corporation, El Cajon, CA, USA). Tube contents were vortexed at the highest speed to grind the tissue, and the ground sample was incubated overnight at 55°. After incubation, samples were centrifuged at 12,000 × *g* for 5 min to precipitate the chitin. RNase A (3 *μ*L at 4 *μ*g/*μ*L) was added to the supernatant and incubated at 37° for 15 min. The supernatant was adsorbed onto a Qiagen column, and the remainder of the preparation followed manufacturer’s instructions. The final volume of sample produced per individual was 200 *μ*L, and DNA was stored at −20° until use. Genomic DNA was submitted to the North Carolina State University Genomic Sciences Laboratory, where it was prepared for sequencing using an Illumina TruSeq LT library preparation kit (Illumina, Inc. San Diego, CA). We barcoded DNA samples from each individual, after which all DNA samples were pooled for sequencing. We sequenced the prepared library on an Illumina NextSeq500 at the North Carolina State University Genomic Sciences Laboratory using 150 base-pair (bp) paired-end reads.

### Read processing and mapping

Sequences were quality filtered to remove all reads with more than 30% of bases having a quality score below Q20 using NGS QC Toolkit v. 2.3.3 (Patel and Jain 2012). Low-quality ends (<Q20) were trimmed from the 3′ end of remaining reads to improve overall alignment quality (Del Fabbro *et al.* 2013). Remaining filter-trimmed reads were mapped to the *H. zea* reference genome (Pearce *et al.* 2017) with Bowtie v. 2.3.2 (Langmead *et al.* 2009) in end-to-end mode using the highest sensitivity preset parameters (–very-sensitive). Alignment files were cleaned to keep only reads in proper pairs with robust mapping quality (MAPQ ≥ 10) using SAMtools v. 1.5 (Li 2011), and PCR and optical duplicates were identified and removed using Picard v. 2.10.5 (http://broadinstitute.github.io/picard). The cleaned alignment files were used to call SNPs with SAMtools v. 1.5 using the mpileup function, and SNP and indel genotypes in Variant Call Formatted (VCF) files were generated using BCFtools. The VCF files were filtered prior to population genomic analysis to only include loci that were: 1) genotyped in at least 50% of individuals, 2) were sequenced to a minimum depth of coverage of 5 and maximum of 2.5 times the mean genome-wide coverage, 3) had a minor allele frequency (MAF) of > 0.1, and 4) were biallelic, using VCFtools v. 0.1.15 (Danecek *et al.* 2011).

### Genetic diversity

Following genotype quality filtering, levels of genetic diversity within GA and GA-R were estimated using various metrics. We estimated the proportion of polymorphic sites (P_N_), and average MAF with PLINK v.1.07 (Purcell *et al.* 2007). We thinned our dataset to 1 SNP per 5kb to reduce the number of SNPs in linkage disequilibrium in our dataset, and then calculated observed (H_O_) and expected (H_E_) heterozygosity and inbreeding coefficients (*F*_IS_) for each line using the hierfstat package (v. 0.04-22, Goudet 2005) in R (v. 3.5.3; R Development Core Team 2008). To estimate overall within population genetic diversity, we calculated Nei’s pairwise genetic distance (*d_X_* and *d_Y_*) using the StAMPP package (v. 1.5.1; Pembleton *et al.* 2013).

### Identifying selective sweeps

To identify putative selective sweeps, we searched for regions of the genome with high divergence between GA and GA-R and exceptionally low genetic variation in GA-R, as measured by heterozygosity. We calculated the heterozygosity of within-population pools of individuals (Hp; Rubin *et al.* 2010) using 40-kb windows with 20-kb of overlap. Hp was calculated as follows:

$$H_{p}=\frac{2\sum n_{MAJ}\sum n_{MIN}}{{\left(\sum n_{MAJ}\sum n_{MIN}\right)}^{2}}$$

where n_MAJ_ is the number of major alleles and n_MIN_ the number of minor alleles in the window. To prevent spurious signals from few SNPs, we excluded windows with fewer than 10 SNPs. According to Rubin *et al.* (2010), we standardized estimates of Hp using a Z transformation (ZHp), and putative regions under selection were identified as being over 6 standard deviations from the mean (ZHp < -6). Using the same sliding window and step size, we also calculated *F*_ST_ to quantify genetic divergence between GA and GA-R in PLINK. Sliding-window averaged *F*_ST_ values were also Z-transformed, although no regions with very low heterozygosity in GA-R (ZHp < -6) also showed *F*_ST_ values greater than 6 standard deviations from the mean *F*_ST_ value calculated for the GA and GA-R comparison. However, some regions of low heterozygosity in GA-R did display *F*_ST_ values greater than 1 standard deviation from the mean (Z*F*_ST_ > 1.1), and for these regions, the *F*_ST_ values ranged from 0.44-0.74.

We used msms simulations (Ewing and Hermisson 2010) to calculate the probability that the observed genetic divergence between strains (*F*_ST_ > 0.44) could have occurred along a 40kb window without selection, given a population demographic scenario similar to what our *H. zea* lines experienced. As a test of sensitivity, additional simulated genotypic datasets were also developed to test population demographic scenarios with smaller population sizes per generation, as well as initial collection sizes. The distributions of *F*_ST_ values were compared among scenarios. The full description of these simulations can be found in File S1.

Finally, we calculated sliding window averaged *F*_ST_ values for the pair of lines using the p*F*_ST_ function in vcflib (https://github.com/vcflib) over a sliding window size of 5-kb with a 1-kb step size. These values were compared with an empirically-derived estimate of genome-wide divergence between GA and GA-R. A Benjamini and Hochberg (1995) false discovery rate (FDR) correction was applied to the p-values for each of these statistical tests. All of these tests taken together indicated that there was one region of the genome where multiple 40kb genomic windows had ZHp values < -6, statistically significant FDR-corrected *F*_ST_ values and Z*F*_ST_ estimates > 1.1, the latter of which appeared unlikely in the absence of selection, according to msms simulations. In this region with the strongest evidence of a selective sweep based on the aforementioned criteria, one of the genes we found was called *cadherin-86C* (*cad-86C*). Because of strong evidence of selection in and around this gene in GA-R, and evidence of cadherin involvement in Bt resistance in other lepidopteran species (Gahan *et al.* 2001; Morin *et al.* 2003; Wang *et al.* 2005; Zhang *et al.* 2017; Wang *et al.* 2018), we examined this region further.

### Verification of heterozygosity and genetic divergence at cad-86C by Sanger sequencing

Further evidence for selection at the *cad-86C* gene in GA-R was gathered using a Sanger sequencing approach. Because our whole genome resequencing data were from 5 individuals per line, we amplified and sequenced two ∼600 base-pair (bp) regions of the targeted gene across 24 new individuals in each line to confirm the evidence for selection. These 600 bp target sequences were 1,222 bp apart in a non-coding region of the gene. Primers were designed with PrimerQuest (www.idtdna.com/Primerquest) and are listed in Table S1. Twenty microliter polymerase chain reactions (PCRs) were conducted using 300 ng of genomic DNA per individual, 0.7uM of each forward and reverse primer, 0.2mM dNTPs, and 4uL 5× GoTaq buffer and 1.25 U GoTaq DNA polymerase (Promega Corporation, Madison, WI, USA) according to the recommended protocol. Cycling conditions included denaturation at 95° for 3 min, followed by 30 cycles of 95° for 30 sec, 60° for 30 sec, 72° for 30 sec and a final elongation step at 72° for 1 min on Bio-Rad T100 Thermal Cycler (Bio-Rad Laboratories, Inc. Hercules, CA, USA). PCR products were cleaned using an ExoAP reaction (see File S2) prior to submission for sequencing. Sanger sequencing was performed using BigDye Terminator v3.1 chemistry (Applied Biosystems) and SNPs were called using PolyPhred (Nickerson *et al.* 1997). Sequences were manually edited to remove low quality nucleotide calls at their ends, as well as to incorporate variant SNPs for heterozygotes using PolyPhred output. Trimmed and edited sequences were aligned using Clustal Omega available through the European Bioinformatics Institute (EMBL-EBI), and estimates of within population genetic diversity (π, θ_W_) and Tajima’s D were calculated using the pegas package (v. 0.11; Paradis 2010) for R. A p-value was also calculated for each Tajima’s test, which indicated whether the D statistic was significantly different from 0. Permutation tests, which were custom-coded in R, were used to identify statistically significant differences in π for GA and GA-R at each *cad-86C* locus.

### Comparison of CAD-86C to other cadherin proteins by sequence alignment

We compared our *H. zea* CAD-86C protein sequence (ID = 537580 from the *H. zea* assembly annotation) with CAD-86C orthologs from other species, as well as other cadherins (BtR and CAD2) known to be involved in Bt resistance. All available cadherin protein sequences from 6 lepidopteran species (*H. zea*, *Pectinophora gossypiella*, *Heliothis virescens*, *Helicoverpa armigera*, *Chilo suppressalis*, *and Bombyx mori*), as well as CAD-86C from *Drosophila melanogaster* were acquired from NCBI. Protein sequences used for analysis, along with their GenBank accession numbers, can be found in File S3. BtR orthologs and the *C. suppressalis* CAD2 were first aligned to our *H. zea* CAD-86C sequence, and a percentage identity matrix was calculated using T-Coffee available through EMBL-EBI (Madeira *et al.* 2019). All available BtR, CAD2, and CAD-86C sequences were then aligned to one another using MUSCLE (Edgar 2004), and a phylogenetic analysis was conducted using the phangorn package (v.2.5.3, Schliep 2011) in R. We used a maximum likelihood approach to distance matrix calculation, assuming a Whelan and Goldman model of molecular protein evolution (Whelan and Goldman 2001). An unrooted phylogeny was produced using a neighbor-joining approach, and bootstrap support values for the tree nodes were calculated using 1000 resampling events.

### Expression of cad-86C in the H. zea midgut

If *cad-86C* were involved in Bt resistance, we reasoned that it should be expressed in the larval midgut. We performed reverse transcriptase quantitative PCR (RT-qPCR) on dissected midgut samples from GA (F81 generation) and GA-R (F62 generation) reared in October 2017. GA-R had been selected for resistance to Cry1Ac in 6 additional non-consecutive generations between the F52 generation used for genomic analysis (see above) and the F62 generation. Resistance to Cry1Ac was verified in dissected GA-R larvae by selecting F62 neonates with a diet overlay bioassay using 40 µg Cry1Ac per cm^2^ of diet surface (Carrière *et al.* 2018a). Susceptibility to Cry1Ac was also verified in dissected GA larvae by selecting F81 neonates with a diet overlay bioassay using a concentration of 30 µg Cry1Ac per cm^2^ of diet surface. Seven days after these bioassays were initiated, GA-R larvae that reached third instar or larger were considered resistant, while GA larvae that remained at first instar were considered susceptible to Cry1Ac. Resistant larvae from GA-R and susceptible larvae from GA were transferred to non-Bt diet and their midguts were dissected upon reaching 4^th^ or 5^th^ instar. Dissections were done in ice-cold RNAlater+PBS mixture. Whole midguts were stored individually in RNAlater for total RNA extraction.

Total RNA from dissected midguts was isolated using a Zymo Direct-zol RNA miniprep according to the protocol recommended by the manufacturer. We verified RNA quality and determined RNA concentration on an Experion automated electrophoresis station. We synthesized first strand cDNA using RevertAid H minus reverse transcriptase and 1µg of total RNA in a 20µL reaction. Gene specific primers were used to amplify *cad-86C* and an endogenous control gene, α-Tubulin (see Table S2 for primer sequences). We performed 20µL qPCR reactions using 10ng of cDNA, 0.5µM primers, and 10uL PowerUp SYBR Green PCR Master Mix (Applied Biosystems). Cycling conditions included initial incubation at 50° for 2 min, followed by denaturation at 95° for 2 min, and then 40 cycles at 95° for 15 sec, 60° for 1 min on a 7300 Applied Biosystems Real Time PCR System. All reactions were run in triplicate for the 13 GA-R and 14 GA individuals. We observed a single peak in the dissociation curves for all reactions, and PCR efficiencies > 90% for each primer pair using LinRegPCR v.2017.1 (Ruijter *et al.* 2009). Gene transcript levels were normalized to α-Tubulin and relative expression was standardized using the gene transcript levels detected in GA individuals. Expression of *cad-86C* in GA-R relative to GA was calculated using 2^-∆∆Ct^.

### Long-read sequencing of cad-86C cDNAs

To analyze the expressed gene product and putative protein sequence, we designed barcoded PCR primers in the 5′ and 3′ UTR regions of *cad-86C*, amplified the complete cDNA, and sequenced this cDNA by single molecule sequencing. The midgut cDNAs from 13 GA-R and 14 GA individuals were subjected to high fidelity PCRs using the barcoded primer sequences found in Table S3. Full length cDNAs were amplified using Q5 High Fidelity DNA polymerase in mastermix format (New England Biolabs Inc., Ipswich, MA, USA). The 50µL reactions contained 25ng cDNA, 0.5µM primers, and 25µL of 2× Q5 mastermix, and cDNAs were amplified under the following cycling conditions: initial denaturation at 95° for 3 min, followed by 30 cycles of 95° for 30 sec, 60° for 30 sec, 72° for 3:40 min and a final elongation step at 72° for 1 min on Bio-Rad T100 Thermal Cycler (Bio-Rad Laboratories, Inc. Hercules, CA, USA). Amplicons were run on a 1% agarose gel and the highest molecular weight fragments (> 5kb) were excised, purified using a Zymoclean Gel DNA recovery kit (Zymo Research, Irvine, CA, USA) following the manufacturer’s protocol, and the quantity of purified cDNA was measured using an Agilent D1000 Screentape System (Agilent Technologies, Inc. Santa Clara, CA, USA). Amplicons were then pooled in equimolar amounts, and sequenced on a single Pacific Biosciences (PacBio) SMRT cell at the North Carolina State University Genomic Sciences Laboratory.

### Identification of CAD-86C amino acid substitutions

A PacBio-generated bam file was converted to fastq file by bedtools (Quinlan and Hall 2010), and sequences from individuals were demultiplexed using the bbduk.sh script from bbmap (Bushnell 2014). Parameters k and restrictleft were set to 18, which was equal to the primers’ lengths, so that the software only looked for primers matching the leftmost 18 bp. Filtering was done by the FASTA manipulation tool of Galaxy (https://usegalaxy.org; Blankenberg *et al.* 2010), and the minimal and maximal length parameters were set to 5000 and 0, respectively, to return all sequences longer than 5000 bp. The correction phase of Canu (Koren *et al.* 2017) was used to improve the accuracy of base calls in PacBio long reads. All error-corrected reads were aligned to the cDNA sequence of *H. zea*’s *cad-86C* gene using the mapPacBio.sh script of bbmap. The results were viewed in Integrative Genomics Viewer (IGV) (Robinson *et al.* 2011).

Consensus cDNA sequences were identified for each GA and GA-R individual, and ambiguous regions with two alleles, indicating an individual was heterozygous at a locus, were manually edited based upon visual inspection of sequences in IGV. Nucleotide sequences were translated to protein sequences using the translate tool in ExPASy (https://web.expasy.org/translate/), and amino acid sequences were aligned to each other with T-Coffee (Notredame *et al.* 2000) to identify potential amino acid substitutions. A two-sided Fisher’s exact test was used to compare the distribution of CAD-86C amino acid variants between lines.

### Data availability

Whole genome sequencing data are available on NCBI under the BioProject ID PRJNA599999. Sanger, qPCR, and PacBio data can be found in Dryad Digital Repository https://doi.org/10.5061/dryad.1c59zw3s1. Scripts used for analysis of PacBio data can be found at https://github.com/rguo1990/Hzea_PacBio_sequencing. Scripts used for all other analyses can be found at https://github.com/mcadamme/GA_and_GAR. Supplemental material available at figshare: https://doi.org/10.25387/g3.11549403.

### Description of figshare contents

Figures S1 and S2 contain the permutation test resampling distributions used to identify statistically significant differences between nucleotide diversity estimates for amplicons of *cad-86C* 1b and 2b primer pairs. Figure S3 is a gel image showing the 5,539 bp *cad-86C* cDNA amplicons from the *H. zea* larval midguts. Table S1 contains the *cad-86C* 1b and 2b primer sequences. Table S2 contains the qRT-PCR primers for *cad-86C* and the α-Tubulin control. Table S3 contains the barcodes used for identification of *cad-86C* sequences from each individual. Table S4 contains a summary of the number of Illumina reads produced per individual, the number that remained after filtering, and coverage depth. Table S5 contains a summary of the genomic regions with low heterozygosity in GA-R and high genetic divergence between GA and GA-R. Table S6 contains a percent identity matrix for the *H. zea* CAD-86C with other cadherin proteins known to be involved in Bt resistance. Table S7 contains a summary of the PacBio sequencing reads produced for each *H. zea* individual, and the number of reads remaining after filtering. Table S8 contains haplotype frequency information for *cad-86C* cDNA sequences. File S1 describes the methods used to run msms. File S2 contains our ExoAP clean protocol. File S3 contains the cadherin sequences used for phylogenetic analysis. File S4 shows the cadherin protein sequences aligned by T-Coffee.

## Results

### WGS data and variant call

A total of 427,136,781 raw PE reads were generated, with 356,202,508 remaining after quality filtering, and 179,040,743 remaining after mapping to the reference genome (Table S4). Uniquely placed reads covered 74.3% of the 335.5 megabases (Mb) genome, with a mean genome wide depth of coverage of 16.1× (range 7.96-23.2×) across all individuals. The initial number of variants before filtering was 5,106,839. After filtering, the total number of SNPs used for population genomic analysis was 1,986,042, and the total number of short indels was 422,149 (2,408,191 total markers).

### Genetic diversity and selective sweeps

**Figure 1:**
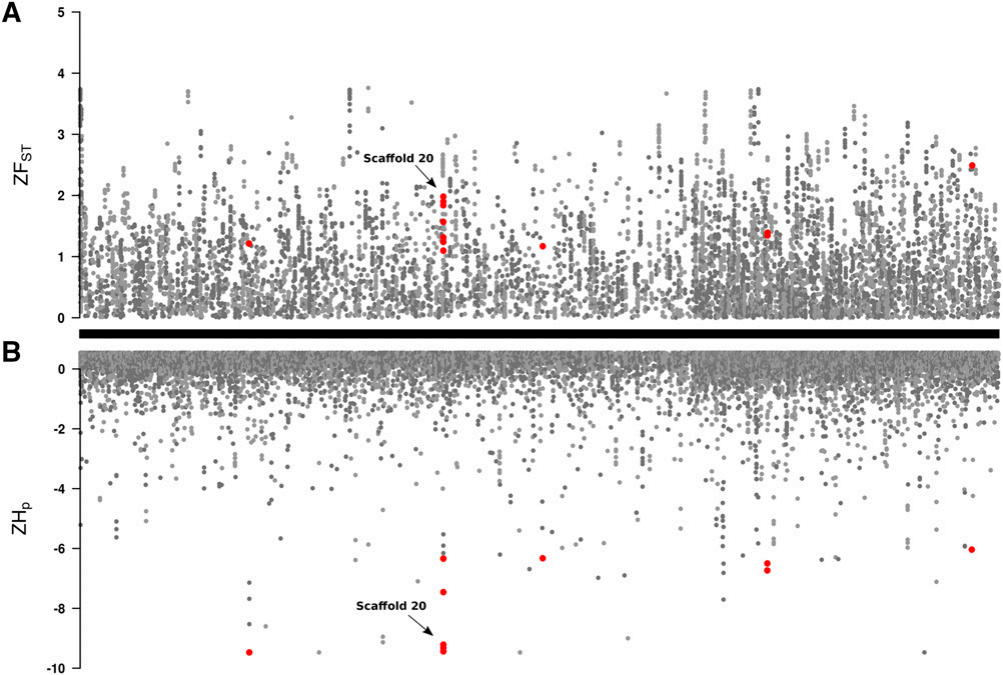
(A) Z-transformed *F*_ST_ values were used to estimate genetic divergence between GA and GA-R in 40-kb sliding windows with a 20-kb step size along the *H. zea* genome. (B) Z-transformed pooled heterozygosity in the GA-R line for the same 40-kb sliding windows with a 20-kb step size. Each of the 2,975 scaffolds that comprise the *H. zea* reference genome are indicated by the alternating light and dark gray points. Genomic windows identified as potentially under selection are in red.

**Figure 2:**
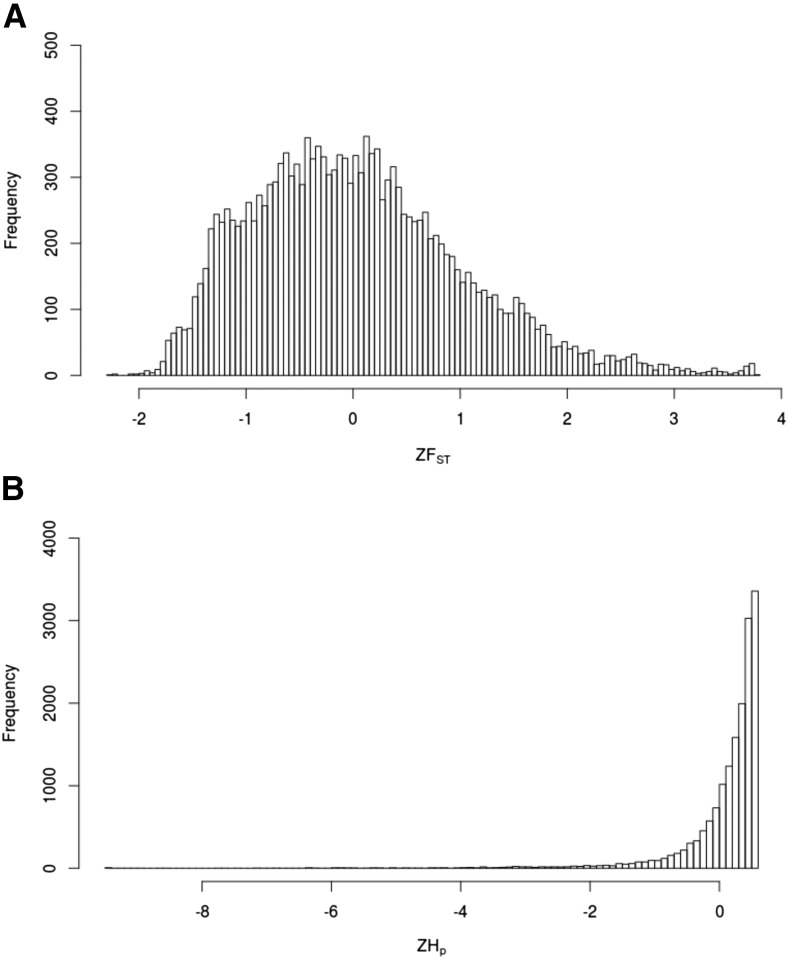
Frequency distributions of (A) Z-transformed *F*_ST_ values for the comparison of genetic divergence between GA and GA-R, and (B) Z-transformed pooled heterozygosity in the GA-R line.

Genome-wide average values of pairwise genetic distance (*d_X_*, *d_Y_*) were similar for GA and GA-R (Table 1) as determined by their overlapping 95% confidence intervals. However, GA-R had significantly higher observed heterozygosity (H_O_), and a significantly lower inbreeding coefficient (*F*_IS_). Overall, the two lines were moderately diverged from each other with a genome-wide *F*_ST_ value of 0.23. Based on heterozygosity estimates among GA-R individuals, a total of 38 genomic windows across 24 scaffolds were identified as putative regions under selection (Figure 1). These candidate regions corresponded to 38 genomic annotations located either upstream or downstream of the identified window (Table S5). The distribution of ZHp values in GA-R indicated a high degree of heterozygosity across most 40-kb windows throughout the genome (Figure 1B). We then examined the Z-transformed *F*_ST_ values across these 40-kb genomic windows, but no 40-kb window had Z*F*_ST_ > 6 (Figure 1A). The maximum Z*F*_ST_ was 3.79 and the Z*F*_ST_ distribution was right skewed (Figure 2A), indicating that there were a number of genomic regions with moderate to high genetic divergence between lines (Figure 1A).

**Table 1:** P_N_ is proportion of polymorphic sites, MAF is the mean minor allele frequency, H_O_ and H_E_ are observed and expected heterozygosity, *F*_IS_ is the inbreeding coefficient, and *d_X_* and *_Y_* are Nei’s average pairwise genetic distance between individuals in a population. Bias-corrected and accelerated bootstrapped 95% confidence intervals (N = 1000) are presented in parentheses. Bolded lines are statistically significant at the *P* < 0.05 level. H_O_, H_E_, *F*_IS_, and *d_X,Y_* were calculated with a thinned SNP dataset of 51,011 SNPs to reduce bias in these estimates associated with linkage disequilibrium

	Susceptible	Resistant
Sample Size	5	5
P_N_	0.738	0.823
MAF	0.225	0.241
**H_O_**	**0.312 (0.310, 0.315)**	**0.347 (0.344, 0.349)**
**H_E_**	**0.329 (0.327, 0.330)**	**0.345 (0.343, 0.347)**
***F*_IS_**	**0.008 (0.004, 0.012)**	**-0.027 (-0.030, -0.024)**
*d_X,Y_*	0.180 (0.140, 0.209)	0.182 (0.137, 0.211)

For those genomic regions displaying low Hp (ZHp < -6) in GA-R, several showed regions with Z*F*_ST_ > 1 between lines. The untransformed *F*_ST_ values across these 40Kb windows ranged from 0.44 - 0.74. Simulations with msms indicated that 40Kb window-averaged *F*_ST_ values of ≥ 0.44 were very unlikely under neutral expectations, given the population demographic conditions under which GA and GA-R were cultured. Under these conditions, none of the 10,000 simulations without selection produced *F*_ST_ ≥ 0.44. Reducing the initial collection size in the simulations from the actual value of 180 to 90 or 45 individuals did not produce *F*_ST_ > 0.44 in 10,000 runs. When the simulated population sizes per generation were reduced to 50 or 25% of those used in our experiment, *F*_ST_ values exceeded 0.44 in two and 130 simulations of 10,000 (*P* = 0.0002 and 0.013), respectively. Thus, even with much smaller population sizes, the probability of producing *F*_ST_ ≥ 0.44 was still low.

**Figure 3:**
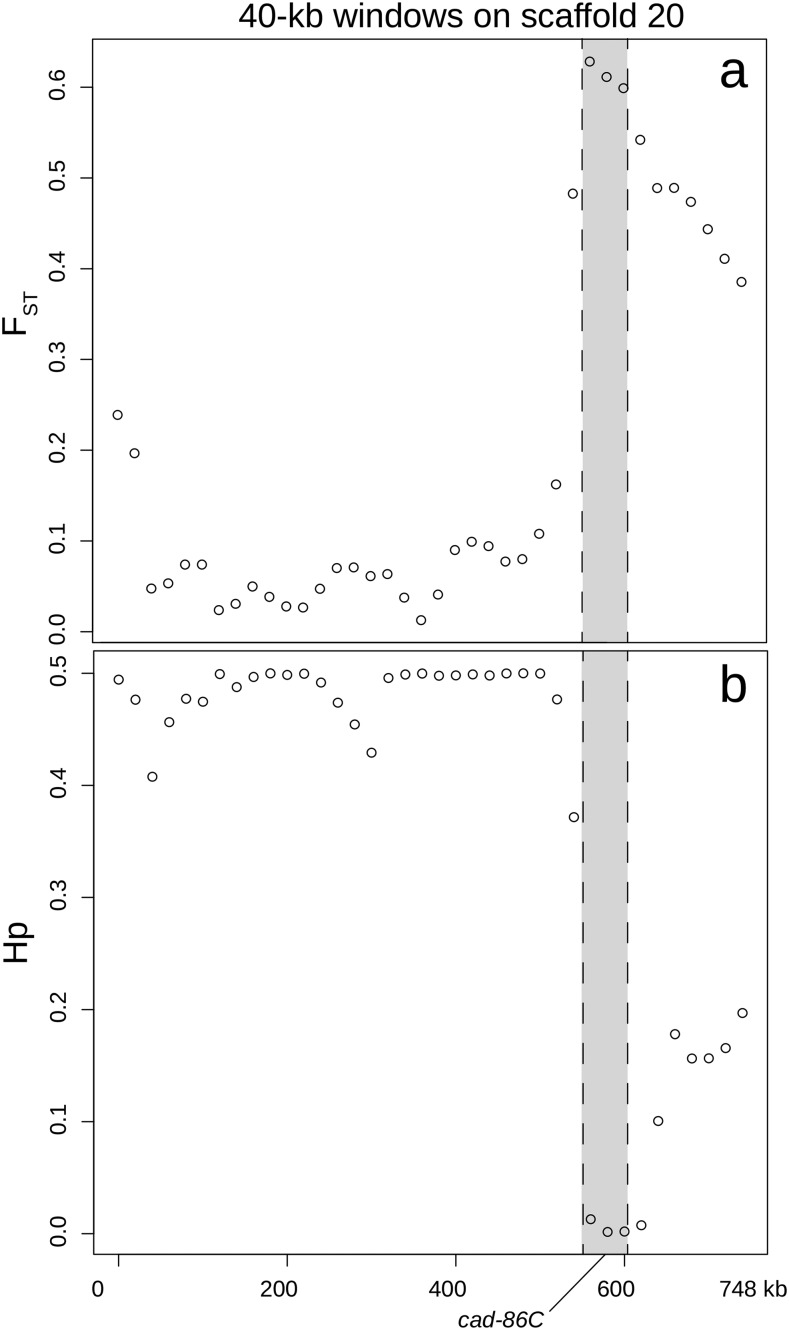
(a) *F*_ST_ between lines and (b) pooled heterozygosity in the GA-R line along 40-kb sliding windows surrounding the putative selective sweep on scaffold 20. *cad-86C* is in gray.

As further confirmation of genomic divergence in these regions, we observed statistically significant *F*_ST_ values (*P* < 0.05) in five of the already identified gene-containing regions using p*F*_ST_, and we identified these as putative selective sweeps (Table S5). These regions were found on five different scaffolds and contained twelve predicted genes: *sol-1* (suppressor of lurcher-1*)*, *cad-86C* (cadherin-86C), *map3k15* (mitogen-activated protein kinase kinase kinase 15), *msp20* (muscle-specific protein 20), *not1* (CCR4-Not complex subunit 1), *cpr47* (cuticular RR1 motif 47), *cgrrf1* (cell growth regulator with RING finger domain 1), *itf46* (intraflagellar transport 46), *cfap100* (cilia and flagellar associated protein 100), *mocs2* (molybdopterin synthase catalytic subunit), *zcchc24* (zinc finger CCHC domain containing 24), and an uncharacterized protein. The longest of these putative sweeps extended from 560,000bp on scaffold 20, just upstream of *cad-86C* (ID537580), through the entire length of *cad-86C*, as well as two other genes (an uncharacterized protein and *map3k15*), and ended at bp 740,000. Although the region was broad, the greatest reduction in heterozygosity in GA-R and greatest divergence (*F*_ST_) between lines overlapped with *cad-86C* (Figure 3), a member of a gene family implicated in Bt resistance in other lepidopteran species (Gahan *et al.* 2001; Morin *et al.* 2003; Wang *et al.* 2005; Zhang *et al.* 2017; Wang *et al.* 2018). This led us to further examine whether *cad-86C* had the potential to serve as target of Cry1Ac selection in *H. zea*.

### Verification of heterozygosity and genetic divergence at cad-86C by Sanger sequencing

Sanger sequencing of 421 and 520 bp within non-coding regions of *cad-86C* revealed 20 and 9 SNPs in GA and 21 and 14 SNPs in GA-R, respectively (n = 22-24 additional individuals per line). The variation in the numbers of SNPs is reflected in the Watterson’s theta values (θ_W_), which were 4.5 and 2.0 for GA, and 4.8 and 3.2 for GA-R, respectively (Table 2). Within population values of π, however, demonstrated that the allele frequency distributions were different between lines. π was always significantly lower for GA-R than GA according to a permutation test (*P* < 0.001; Table 2; Figures S1 and S2), indicating a reduction in intermediate frequency alleles in GA-R. For the 421 and 520 bp sequences, nucleotide diversity (π) values were 0.016 and 0.006 for GA, and 0.009 and 0.004 for GA-R, respectively. Tajima’s D test statistics were negative for GA-R, which could indicate a reduction in genetic diversity by purifying selection or a population contraction. D was positive for GA, which could signal greater genetic diversity than expected due to balancing selection or a recent population expansion. Only one Tajima’s D statistic, calculated with the 520 bp GA sequence data were significantly different from 0 at the α < 0.05 level, however (Table 2).

**Table 2:** Estimates of genetic diversity within the GA and GA-R lines in two non-coding regions of the *cad-86C* gene. N represents the number of chromosomes sampled

Primer Pair	Sequence Length	Line	N	θ_W_	π	Tajima’s D
Cad_1b	421	GA	48	4.5	0.016	1.5
		GA-R	44	4.8	0.009	−0.74
Cad_2b	520	GA	48	2.0	0.006	2.3*
		GA-R	48	3.2	0.004	−0.86

*P* < 0.05 *

### Comparison of CAD-86C to other cadherin proteins by sequence alignment

**Figure 4:**
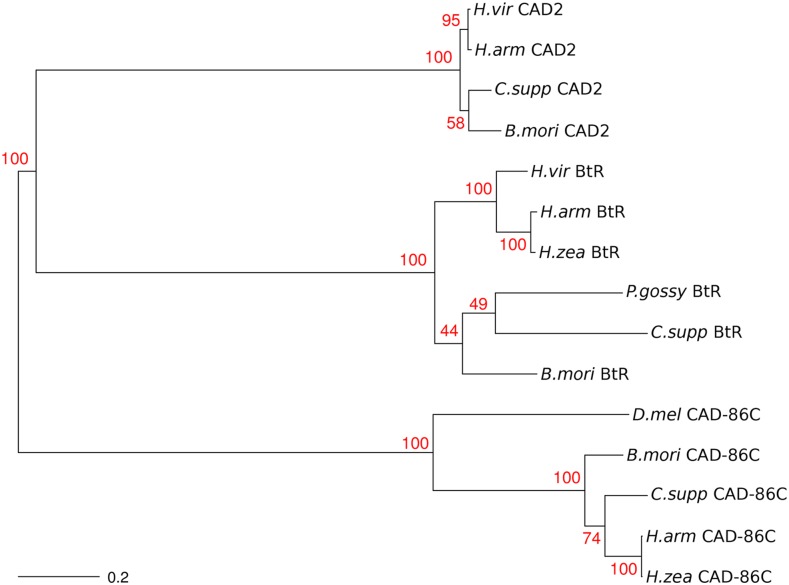
Unrooted neighbor-joining tree indicating the phylogenetic relationships between CAD-86C, CAD2, and BtR. Numbers in red are bootstrap support values (N = 1000) for the tree nodes. A scale bar for genetic distance is in the lower left corner.

Amino acid sequence identity was 56–84% among five cadherin proteins involved in Bt resistance in other Lepidoptera, but only 17–22% between CAD-86C from *H. ze*a and each of these five proteins (Table S6). When we reconstructed the phylogenetic relationships among 14 cadherin proteins, clustering occurred by putative homologs rather than by species (Figure 4). High bootstrap support for the gene clusters demonstrated that CAD-86C was not homologous to other cadherins involved in Bt resistance.

### Expression of cad-86C in the H. zea midgut

Quantitative PCR revealed that *cad-86C* was transcribed in the midgut of GA and GA-R larvae, with 1.6 fold higher expression in GA-R (Mann-Whitney *U*-test, *P* = 0.037). Survival to at least 3^rd^ instar was significantly higher for GA-R (61.3%, N = 3840) as compared to GA (1.0%, N = 512) (Fisher’s exact test, *P* < 0.0001) reared on Cry1Ac treated diet at the time of midgut dissection.

### Identification of CAD-86C amino acid substitutions

A 5,539 base pair PCR amplicon, which corresponded to the *cad-86C* coding sequence, was produced for most GA and GA-R individuals (Figure S3). Following PacBio sequencing of these amplicons, a total of 1,081,073 PacBio long reads were generated from 30 *H. zea* individuals. After demultiplexing and quality and length filtering, 14,862 reads remained (minimum length = 5000 bp, Table S7), and 22 individuals (11 per line) were used for analysis.

Using 11 individuals from each line (total n = 22), we identified five predicted CAD-86C protein variants (File S4). The distribution of these variant sequences differed significantly between GA and GA-R (Fisher’s exact test, *P* = 0.002; Table 3). Variant 1, the full-length reference protein, was the most common variant in both lines, accounting for 86% of the sequences for GA-R (nine homozygotes and one heterozygote), and 50% for GA (five homozygotes and one heterozygote (Tables 4 and S8). Variants 2 and 3, which occurred in only one individual from GA-R, were identical to the reference sequence except for an insertion at 600,850-600,883 bp of scaffold 20. This insertion introduced a premature stop codon, and was expected to yield a truncated protein. Variant 4, which accounted for 41% of the sequences for GA and 5% for GA-R, had a 15 bp deletion (5′- GAT AAT ACT GCA ACA - 3′) at position 599,832-599,846 bp, and a SNP at 603,408 bp. The deletion was expected to cause the loss of a 5 amino acid sequence (DNTAT) from cadherin repeat domain five, part of the extracellular protein domain which is proximal to the membrane-spanning region. The SNP was expected to produce a threonine to lysine substitution as compared to the reference (T1706K). Variant 5, which appeared in only one individual from GA, had the same 15 bp deletion present in variant 4, and an extra exon in position 600,522-600,574 bp that would terminate the amino acid sequence, producing a truncated protein.

**Table 3:** CAD-86C amino acid sequence variant counts for the 22 *H. zea* individuals from the GA and GA-R lines. Counts represent chromosomes sampled (n = 2 per individual)

Variants	GA	GA-R
1	11	19
2	0	1
3	0	1
4	9	1
5	2	0

Upon identification of these variants by midgut cDNA sequencing, we revisited our WGS data. All five GA-R individuals were homozygous for the reference sequence at positions 599,832-599,846 and 603,408, and were expected to produce a DNTAT+T haplotype. By contrast, only one individual from GA was homozygous for this haplotype. Two GA individuals were homozygous for the 15 bp deletion leading to the loss of the DNTAT amino acid sequence from the expressed protein, and bore the SNP that caused a threonine to lysine substitution at amino acid position 1706. The final two individuals were heterozygous bearing one copy of the reference allele and the alternate allele that produced the DNTAT deletion. This gave a DNTAT + T haplotype frequency of 1 in GA-R, and 0.4 in GA, which was consistent with our PacBio sequencing data.

## Discussion

Here we used whole genome sequencing to identify putative genomic targets of Cry1Ac selection in laboratory-reared *H. zea*, an important agricultural insect pest. This approach has been used previously to identify signatures of selection in other laboratory-selected insects (Izutsu *et al.* 2012; Jha *et al.* 2015; Ding *et al.* 2018). Reduced heterozygosity in an otherwise heterozygous selected population, combined with increased genetic divergence between selected and unselected lines, serves as a signal that a region of the genome was under selection. Because of small population size, genetic drift often occurs among laboratory-reared lines (Schoen *et al.* 1998; Fritz *et al.* 2016). When drift occurs, loss of heterozygosity and random fixation of alleles can lead to heightened genome-wide divergence between lines, which can impede separating effects of drift and selection (Hahn 2019). As recommended by Chandler *et al.* (2013), we backcrossed GA-R to its ancestral line (GA) and reselected for Cry1Ac resistance. To counteract the effects of drift, we crossed the two subsets within each line every second or third generation. We found a slight, but statistically significant, increase in the genome-wide heterozygosity for GA-R (H_O_ = 0.35) relative to GA (H_O_ = 0.31), which was not observed in a pair of laboratory-selected Bt resistant (YHD2) and susceptible (YDK) *H. virescens* lines maintained without backcrossing (Fritz *et al.* 2016). Furthermore, genome-wide divergence between lines was lower for GA and GA-R (*F*_ST_ = 0.23, n = 5 per line) than YHD2 and YDK (*F*_ST_ = 0.28, n = 43-46 per line). Even so, the genetic divergence between GA and GA-R was slightly higher than that recommended by Pérez-Figueroa (*F*_ST_ = 0.2) for a genome scan to identify adaptive loci (Pérez-Figueroa *et al.* 2010). Therefore, we quantified the extent of genetic divergence (*F*_ST_) between lines for regions of lowest heterozygosity in GA-R.

We detected 5 genomic regions with combined ZHp < -6 and Z*F*_ST_ values >1.1. These regions contained 12 genes, several of which drive various aspects of animal behavior (Table S5). For example, *sol-1* encodes a product that is required for proper function of an ionotropic glutamate receptor, which regulates synaptic transmission and ultimately locomotory behavior in *Caenorhabditis elegans* (Zheng *et al.* 2004). *Muscle-specific 20*, another potential locomotory target of selection, is expressed in the muscle tissue of insect larvae and is likely involved in actin binding (Ayme-Southgate *et al.* 1989). *Map3k15* is a gene from a family involved in the regulation of other genes and ultimately cellular responses to external environmental stimuli (Treisman 1996). Finally, *cfap* genes are thought to play a role in the motility of cilia, such as those on type-1 sensory neurons in *Drosophila*. Ciliary defects are thought to impact a number of sensory processes (*e.g.*, touch, coordination, taste, olfaction and hearing; Jana *et al.* 2016).

Previous studies indicate that some species of Lepidoptera show distinct behaviors when exposed to plant tissue or diet treated with Cry proteins as compared to those exposed to untreated tissue or diet. For example, increased locomotor activity and ballooning behaviors are thought to be the result of larval toxin detection and avoidance (Benedict *et al.* 1992; Men *et al.* 2005; Prasifka *et al.* 2009; Goldstein *et al.* 2010; Ramalho *et al.* 2014). Furthermore, in binary choice tests, larvae from GA-R preferred to feed on a nutritionally optimal Cry1Ac diet relative to a non-Bt suboptimal diet, while larvae from GA consumed more of the suboptimal non-Bt diet (Orpet *et al.* 2015b). This suggested that detection and consumption of a nutritionally optimal diet supersedes toxin avoidance behaviors in GA-R. Genes involved in sensory and locomotory function may have served as targets of Cry1Ac selection in GA-R, conferring these behavioral changes. Future work on the function of genes linked to animal behavior identified here could provide clues as to how behavioral changes occurred in GA-R as a result of Cry1Ac selection.

Much of our analysis focused on a cadherin gene, *cad-86C*, because disruption of the coding sequence of cadherin proteins or reduced cadherin gene expression confers resistance to Cry1 toxins in other Lepidoptera (Gahan *et al.* 2001; Morin *et al.* 2003; Xu *et al.* 2005; Fabrick *et al.* 2014; Zhang *et al.* 2017; Wang *et al.* 2018). Moreover, this gene had high levels of genetic divergence between lines (*F*_ST_ = 0.44-0.63) and the second lowest heterozygosity estimates in GA-R in the entire genome (Table S5; Figure 1). Simulations with msms using a realistic population demographic scenario under neutral expectations indicated that *F*_ST_ values > 0.44 were not likely to occur due to drift alone (*P* < 0.0001). Furthermore, our Sanger data indicated that GA-R *cad-86C* sequences always had significantly lower π values than did GA, opposite the overall genome-wide trends in other measures of genetic diversity for these two populations. Tajima’s D values also trended in opposite directions, with positive values associated with GA and negative values associated with GA-R. At one 520 bp *cad-86C* sequence, the GA Tajima’s D statistic was significantly higher than zero, indicating that there was higher than expected genetic diversity in GA at *cad-86C*, which was also opposite genome-wide trends for these two populations. Taken altogether, these results indicated that distinct evolutionary processes were likely occurring at this gene for the two populations.

The *H. zea* CAD-86C protein sequence showed only 17–21% identity with other cadherin proteins involved in Bt resistance (Table S6). As far as we know, the function of CAD-86C has been described only for embryonic organogenesis in *D. melanogaster* (Lovegrove *et al.* 2006; Fung *et al.* 2008; Schlichting *et al.* 2008). To demonstrate that *cad-86C* has a potential role in Bt resistance, we quantified and compared transcription of this gene in the midguts of GA and GA-R larvae. This was of particular interest because of the role of midgut-expressed cadherins other Lepidoptera (Gahan *et al.* 2001; Morin *et al.* 2003; Wang *et al.* 2005; Zhang *et al.* 2017; Wang *et al.* 2018). The 1.6-fold higher expression of *cad-86C* in GA-R relative to GA was statistically significant, but it seemed unlikely this was the primary cause of the >10-fold difference in resistance between these lines (Orpet *et al.* 2015a). Therefore, further experiments quantified the difference between lines in the frequency of predicted protein sequence variants.

We found that the presence of a 15bp indel, whose *in silico* translation produced an amino acid sequence DNTAT, was at a frequency of 0.5 in GA and 0.95 in GA-R according to PacBio sequencing of 22 individuals (n = 11 per line; Table 3; Figure S4). These frequencies included GA-R individuals with amino acid variants 2 and 3, which also contained the 15bp insertion relative to GA variant 4. The same trend was present in individuals from each line sequenced by Illumina short reads. Under one possible evolutionary scenario, the DNTAT haplotype was present as a standing genetic variant in the original field-collected population from Georgia, and increased in frequency under the Cry1Ac selection regime experienced by GA-R, but remained at lower frequency in the unselected GA.

Interestingly, the DNTAT mutation was found in the cadherin repeat domain that was most proximal to the membrane-spanning region of the protein. Previous investigations of BtR indicated that the repeat domain most proximal to the membrane-spanning protein domain was critical for toxicity, and mutations in this region promoted Cry1Ab resistance (Hua *et al.* 2004). If *cad-86C* were involved in Cry1A toxin binding, a mutation of this nature may reduce toxin binding and activation, ultimately leading to resistance in GA-R. The putative protein-coding changes, in concert with evidence of midgut gene expression, and the breadth of the selective sweep identified by our whole genome sequencing data analysis, provide compelling evidence for this evolutionary scenario, and suggest that this gene is under selection by Cry1Ac in GA-R.

There are a few caveats to interpretation of our findings, however. Given the nature of our experiments, it is difficult to infer the directionality of the change in allele frequency experienced by GA and GA-R after the lines were established in the laboratory. A second evolutionary scenario, distinct from the one described above, is that the DNTAT mutation was already at high frequency in the moderately resistant founder population when they were collected from Cry1Ab-expressing corn in 2008 (Brévault *et al.* 2013). Due to experimentally imposed Cry1Ac selection, the DNTAT mutation may have been maintained at high frequency in GA-R, but declined in the GA line reared on untreated diet. Many insecticide resistance mutations reduce fitness in the absence of insecticides (Kliot and Ghanim 2012), and Bt resistance mutations are no exception (Gassmann *et al.* 2009; Carrière *et al.* 2018b). The changes in allele frequency described under this scenario could have occurred if the Cry1Ac resistance-conferring mutation was costly to maintain in the absence of selection. Previous work has indicated that the fitness cost of resistance mutations in GA-R is not high enough to cause rapid loss of a resistance phenotype when selection is removed for 1-2 generations (Carrière *et al.* 2018a). GA and GA-R survival and development times were similar on untreated artificial diets with various casein:sucrose ratios, and survivorship, pupal weight and development time did not differ on young non-Bt cotton plants (Carrière *et al.* 2018a 2019). Nonetheless, fitness components were lower in GA-R in the absence of Bt toxins under certain conditions. Pupal weight, for example, was generally lower for GA-R than GA on untreated artificial diet (Orpet *et al.* 2015a). Furthermore, survival was lower for GA-R larvae fed upon old non-Bt cotton plants as compared to GA larvae (Carrière *et al.* 2018a). Such fitness costs, if present in the ancestral population of GA and GA-R, could underlie the decline in resistance allele frequency described for GA in this second evolutionary scenario.

Identification of a selective sweep which includes *cad-86C* or any one of the other 11 genes we identified in our study, cannot demonstrate that they are directly involved in Cry1Ac resistance. Instead, some or all of these genomic regions may be under linked or even indirect selection. It is interesting that the DNTAT amino acid sequence is present in *H. armigera* CAD-86C reference sequences used in our analyses, as well as in the reference genome of *H. zea*. This suggests that the mutation is not novel, but was present in the common ancestor of these two species. Furthermore, both *H. armigera* (Tabashnik & Carrière 2017), and the strain of *H. zea* used for genome sequencing (Pearce *et al.* 2017) are generally considered to be susceptible to Cry1A toxins. Therefore, a third evolutionary scenario may be that the DNTAT mutation is not itself the target of selection, but instead is physically linked to an unidentified but nearby target of Cry1A selection. A fourth evolutionary scenario may be that genes in our identified putative sweep regions, including *cad-86C*, allow for recovery of fitness in individuals bearing mutations with otherwise deleterious effects. Perhaps selection for a mutation that conferred resistance in GA-R was followed by selection for mutations that improve the fitness of individuals bearing such a mutation. In any case, our approach cannot quantify the phenotypic impact of individual mutations on a trait itself. Rather, by comparing the genome-wide changes in allele frequency between the two lines, we can detect signals that indicate selection has taken place.

Finally, there are inherent tradeoffs to using laboratory-selected *vs.* field-selected populations to identify genes under selection (Ffrench-Constant 2013). On the one hand, laboratory-selected populations can provide powerful insights into the genetic mechanisms underlying resistance (Gahan *et al.* 2001), and some Bt resistance mutations identified in laboratory-selected populations have been found in field-selected populations (Zhang *et al.* 2012; Jin *et al.* 2018; Mathew *et al.* 2018; Wang *et al.* 2019). On the other hand, some mutations or molecular mechanisms that produce resistant phenotypes in the lab cannot be found in field-collected individuals (Zhang *et al.* 2012; Fabrick *et al.* 2014; Mathew *et al.* 2018), or even other lab-selected strains. As one example, Caccia *et al.* (2012) determined that increased alkaline phosphatase levels in the midguts of laboratory-selected *H. zea* strain AR1 were associated with Cry1Ac resistance. Yet this mechanism did not explain the resistance in GA and GA-R relative to a susceptible strain (Zhang *et al.* 2019). Instead, changes in the expression of midgut proteases were responsible for resistance to Cry1Ac in both GA and GA-R relative to the susceptible strain, but not for the 14-fold increase in Cry1Ac resistance for GA-R relative to GA.

Here, we further examined GA and GA-R to identify the genes under selection by Cry1Ac, and to determine the molecular mechanisms underlying this 14-fold higher Bt resistance in GA-R. We identified several regions of the GA-R genome with signatures of selection, including one that overlapped with a novel midgut-expressed cadherin. Future work should quantify the phenotypic impacts of *cad-86C* variants in this pair of lines, as well as evaluate the importance of these variants to Cry1A resistance in field-selected *H. zea*.
